# Prevalence and characterization of maxillary sinus septa in a brazilian population

**DOI:** 10.4317/jced.56467

**Published:** 2021-07-01

**Authors:** Deusa-Maria-Mendes Furtado, Paulo-Antônio Martins-Júnior, Tatielly-Karine-Costa Alves, Rafael-Pereira-da Mata Santos, Danielle-Carvalho-Oliveira Coutinho, Idalísio-Soares-Aranha Neto, Bruno-César-Ladeira Vidigal, Guilherme-Augusto-Alves de Oliveira, Micena-Roberta-Miranda Alves e Silva, Flávio-Ricardo Manzi

**Affiliations:** 1 DDS. Department of Dentistry of the Federal University of Minas Gerais. Faculty of Dentistry, Federal University of Minas Gerais. Universidade Federal de Minas Gerais, Avenida Presidente Antônio Carlos, 6627, Pampulha, Belo Horizonte, Minas Gerais, Brazil; 2 PhD. Department of Pediatric Dentistry of the Federal University of Minas Gerais. Faculty of Dentistry, Federal University of Minas Gerais. Universidade Federal de Minas Gerais, Avenida Presidente Antônio Carlos, 6627, Pampulha, Belo Horizonte, Minas Gerais, Brazil; 3 DDS. Department of Dentistry of the Pontifical Catholic University of Minas Gerais. Graduate Program in Dentistry of the Pontifical Catholic University of Minas Gerais. Pontifícia Universidade Católica de Minas Gerais - Departamento de Odontologia Avenida Dom José Gaspar, 500, Coração Eucarístico, Belo Horizonte, Minas Gerais, Brazil; 4 Graduate Program in Dentistry of the Pontifical Catholic University of Minas Gerais. Pontifícia Universidade Católica de Minas Gerais - Departamento de Odontologia Avenida Dom José Gaspar, 500, Coração Eucarístico, Belo Horizonte, Minas Gerais, Brazil; 5 PhD. Department of Dentistry of the Federal University of Minas Gerais. Faculty of Dentistry, Federal University of Minas Gerais. Universidade Federal de Minas Gerais, Avenida Presidente Antônio Carlos, 6627, Pampulha, Belo Horizonte, Minas Gerais, Brazil; 6 MSc. Department of Dentistry of the Pontifical Catholic University of Minas Gerais. Graduate Program in Dentistry of the Pontifical Catholic University of Minas Gerais. Pontifícia Universidade Católica de Minas Gerais - Departamento de Odontologia Avenida Dom José Gaspar, 500, Coração Eucarístico, Belo Horizonte, Minas Gerais, Brazil; 7 PhD. Department of Morphology of the Federal University of Minas Gerais. Faculty of Dentistry, Federal University of Minas Gerais. Universidade Federal de Minas Gerais, Avenida Presidente Antônio Carlos, 6627, Pampulha, Belo Horizonte, Minas Gerais, Brazil; 8 PhD. Department of Dentistry of the Pontifical Catholic University of Minas Gerais. Graduate Program in Dentistry of the Pontifical Catholic University of Minas Gerais. Pontifícia Universidade Católica de Minas Gerais - Departamento de Odontologia Avenida Dom José Gaspar, 500, Coração Eucarístico, Belo Horizonte, Minas Gerais, Brazil

## Abstract

**Background:**

The aim of this study was to assess the anatomic aspects of the maxillary sinus septa, by means of computed tomography images, in a Brazilian population. The results might be of clinical significance in sinus lift surgery planning.

**Material and Methods:**

In the study, 123 computed tomographs obtained from a private radiology clinic were used. They were analyzed by a single, trained and calibrated observer in order to evaluate the presence, quantity, localizations, dimensions, orientations and different characteristics in dentate, partially edentulous and completely edentulous individuals of the sinus septa.

**Results:**

Of the individuals analyzed, 26% had a septum in the maxillary sinus, with 59.6% being classified as complete, 44.2% showed prevalence for the middle region of the maxillary sinus. There was no statistically significant difference between the right and left sides (*p*>0.05).

**Conclusions:**

The majority of patients in the sample analyzed presented to septa in the maxillary sinuses, and when septa were present, a higher number of occurrence was noted in the middle region of the maxillary sinus. (This region is normally the choice of sinus lift surgery). There was no predilection relative to age, sex and type of dentition.

** Key words:**Multislice computed tomography, maxillary antrum, anatomic variation.

## Introduction

The maxillary sinus is a structure normally evaluated by the dental surgeon at the time of surgical planning for rehabilitation with implant supported dental prostheses. The distance from the maxillary sinus floor to the apices of the roots of teeth is longer for the maxillary first premolar and shorter for the mesio-vestibular root of the maxillary second molar (1). These sinuses may present innumerable anatomic variations, with the most common being pneumatization of this cavity into the alveolar process, which occurs in 80% of patients. The maxillary first and second molars showed a higher prevalence of alveolar domes, particularly on the vestibular roots, followed by the third molars and second premolars (2,3).

Within the maxillary sinus it is possible to observe the presence of a structure known as the maxillary sinus septum, or sinus septa (4,5). This structure is defined as consisting of thin cortical bone walls present inside the maxillary sinus, varying in number, thickness and length, originating from the inferior and lateral walls of the maxillary sinus, capable of dividing it into two or more cavities. The sinus septa present variations in different world populations (6). Direction of the septum may be influenced by growth of the maxilla and palatine bone, and this structure may be found in any region of the maxillary sinus, irrespective of the patient’s degree of edentulism (7).

In implant dentistry, to ensure that there is sufficient bone height in the maxilla for the placement of dental implants in the maxillary sinus region, the maxillary sinus lift procedure is performed. The septum may lead to the sinus membrane thickness being smaller in this region, increasing the risk of perforation of the membrane during the surgical procedure. Imaging evaluation of the region is imperative whenever any surgical intervention is to be made in the area. Therefore, knowing about both the normal aspects and presence of anatomical variations in the maxillary sinuses are critical elements for successful maxillary sinus lift surgery and for diminishing the risk of sinus membrane perforation and post-surgical complications (7-14).

Thus, the aim of this study was to analyze the aspects with reference to the maxillary sinus septa (presence, quantity, dimensions, orientation and localization) in multislice computed tomography images, in a Brazilian population. The data of this study will be of great use in approaches to the maxillary sinuses and will provide useful information for adequate and safe surgical planning.

## Material and Methods

This retrospective cross-sectional study was conducted after approval from the local Ethics Committee (CAE: 40961315.0.0000.5137). The study sample was composed of 123 Multislice computed tomography images of the regions of the sinuses of the face bones, obtained from the database of a private dental imaging exam service, in a population of Brazilian nationality, from the city of Belo Horizonte, in the state of Minas Gerais. All of the computed tomographic images were acquired by means of a Multislice Somatom Sensation 64 tomograph (Siemens Healthcare, Erlangen, Germany).

As criteria for inclusion in the sample, the tomographic exams had to be of the sinuses of the face presenting the entire extension of the maxillary sinuses, with adequate visualization of both the right and left maxillary sinuses. The exclusion criteria were exams with the presence of artefacts and/or exams with low quality images.

**Figure 1:**
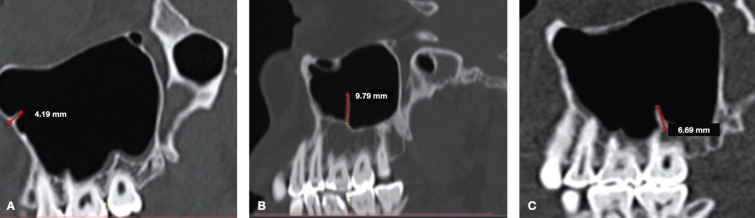
Tomographic images in sagittal cuts showing regions of septa: A) anterior region; B) middle region; C) posterior region.

The septa were classified according to side, position, region and characterization (complete or incomplete). The region of the septum was defined as anterior (distal from the canine to distal from the second premolar), middle (mesial from the first molar to distal from the second molar), and posterior (distal from the second molar to the region of the maxillary tuberosity) (15) (Fig. 1).

**Figure 2:**
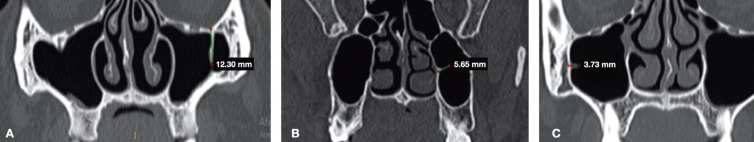
Tomographic images in frontal/coronal cuts showing position of septa: A) superior position; B) middle position; C) lateral position.

In this study, the position of the maxillary sinus septum in relation to the three-dimensional space of the maxillary sinus was also verified. In this context, the septa could be localized in the superior, middle and lateral positions (Fig. 2.

All of the images were evaluated by 2 dentists, specialists in dental radiology and diagnostic imaging, after having been duly trained and calibrated. This study used a computer that contained a GeForce 9500 GT graphics card (Nvidia Corporation, Santa Clara, CA, USA) and an LED LG Flatron E2241 monitor (LG Electronics, Greater Noida, Uttar Pradesh, India) with a resolution of 1920×1080 pixels, together with brightness and contrast levels of the monitor set to their pre-defined conFigurations. 

The data were digitized and organized in the software Statistical Package for Social Sciences (SPSS for Windows, version 20.0, SPSS Inc. Chicago, III, USA). Statistical analyses involved the distribution of frequency and test of association. The Chi-square test was used to compare sex, age and dentition in relation to absence and presence of septa. In addition the Kolmogorov-Smirnov normality tests (sample>50) were performed to verify whether the data were normally distributed. Values of *p*<0.05 were considered statistically significant.

## Results

Of the 123 tomographs of the paranasal sinuses, 54.5% of the individuals were of the female sex and 45.5% of the male sex, with a mean age of 47 years (minimum of 18 and maximum of 90 years). The individuals were divided according to dentition: complete (30.9%), partially complete (57.7%) and edentulous (11.4%) (Table 1).

**Table 1:**
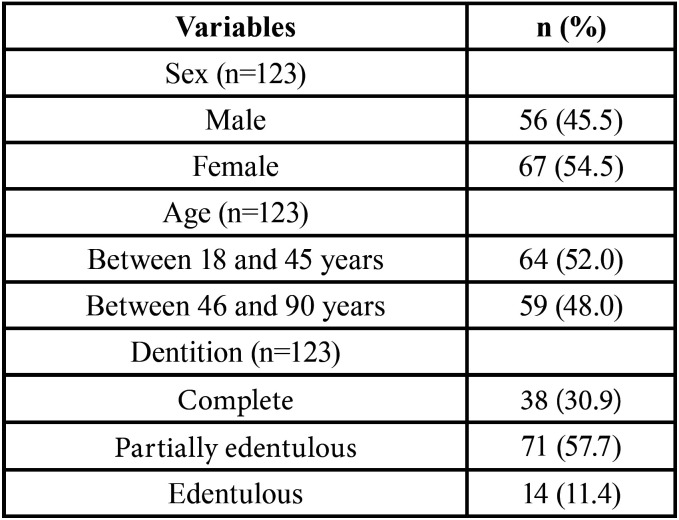
Characteristics of the study population according to sex, age and dentition.

The majority of the individuals evaluated (73.2%) were observed to present no maxillary septa. Relative to side, 13 (36.1%) were localized on the right side, 9 (25.0%) on the left side and 14 (38.9%) were bilateral, without statistically significant difference. By means of descriptive analysis, the majority of septa were found in the superior position; approximately 59.6% of these septa crossed the sinus completely, and 40.4% of the septa crossed the maxillary sinus incompletely. When the septum was present, no significant difference between the right and left maxillary sinuses was found. Analysis of the anatomic region of the maxillary sinus in turn, revealed that 44.2% of the septa showed higher prevalence in the middle region of the maxillary sinus (first and second molars) (Table 2). 

**Table 2:**
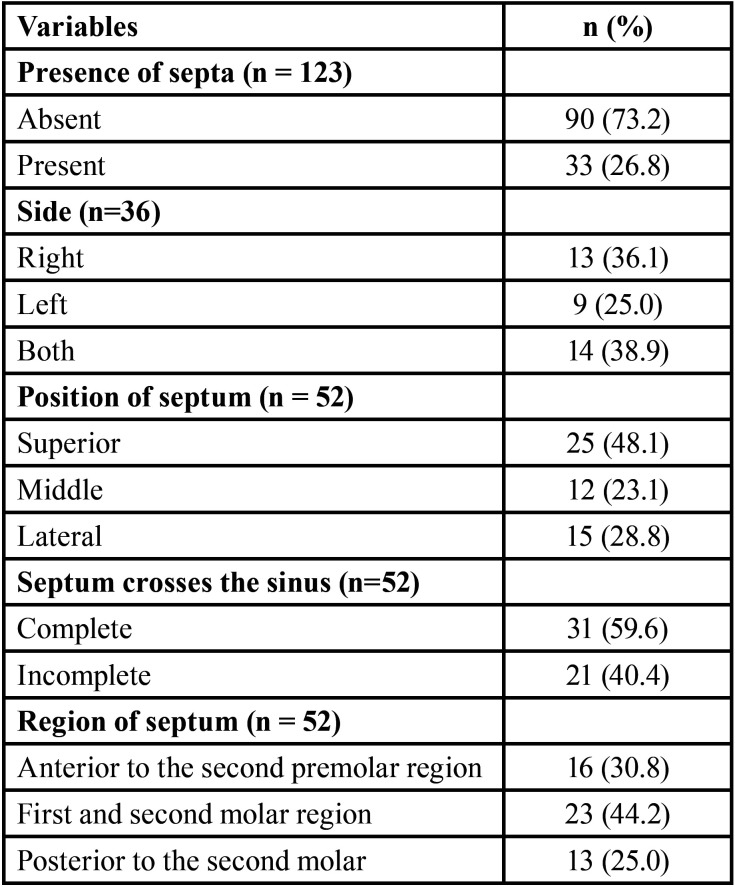
Distribution of septa relative to presence, side, position, complete or incomplete extension, and region of occurrence.

The extension of the septa were measured in three sections: frontal, sagittal and axial, in both the right and left side maxillary sinuses. The data obtained for the septa in the right maxillary sinus showed mean values of 7.7 mm in the frontal section, 7.6 mm in the axial, and 8.7 mm in the sagittal section. Whereas, for the septa in the left maxillary sinus, the mean values were 6.4 mm in the frontal section, 6.9 mm in the axial, and 7.2 mm in the sagittal section (Table 3).

**Table 3:**
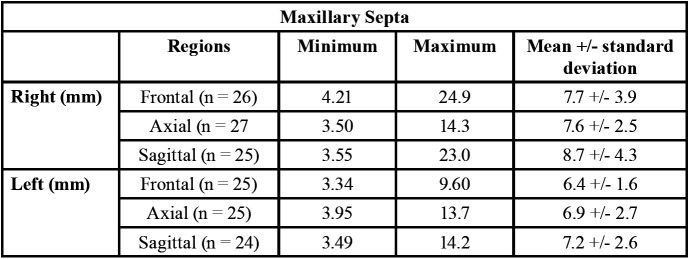
Measurement of maxillary septa in different cuts: frontal, axial and sagittal of the right and left maxillary sinuses.

The results revealed that a total of 33 different individuals presented at least one septum. Of these, 17 individuals presented only 1 septum, 14 had two septa, 1 had 3 septa and 1 individual had 4 septa. By means of descriptive analysis as regards patients of the male sex, 39 (69.6%) had no septum, 8 (14.2%) patients had 1 septum, 8 (14.2%) had 2 septa and 1 (1.7%) patient had 3 septa. For the female sex, 51 (76.1%) patients presented no septa, 9 (13.4%) patients had 1 septum, 6 (8.9%) had 2 septa and 1 (1.4%) patient had 4 septa.

The data in Table 4 revealed statistical significance between the presence and absence of maxillary septa relative to the type of dentition. In general, the septa were noted to be absent in all types of dentition (Table 4).

**Table 4:**
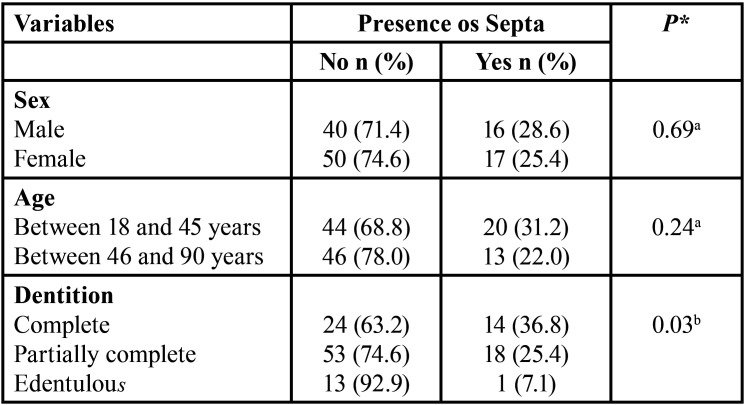
Analysis of the presence and absence of maxillary septa according to sex, age and dentition. aChi-square test, bChi-square for linear trend. Statistical Significance for *p*<.05.

## Discussion

Panoramic radiography has a low level of reliability for detecting maxillary septa (14), whereas, computed tomography provides valuable information about the presence and localization of these structures, because they can be evaluated in all planes, as was done in the present study. According to Maestre-Ferrin *et al*., computed tomography was an effective method for analyzing pre-operative planning of maxillary sinus lift procedures (16). Panoramic radiographs might not be sufficient for exhibiting anatomic variations in the maxillary sinus (16-24).

According to the analyses made in the present study, there was no statistically significant difference relative to their distribution in the different regions, in spite of the majority of them being located in the middle region of the maxillary sinus. Lang and Schulz obtained a similar result, in which 31 septa were present in a total of 106 maxillary sinuses (17). Other studies have revealed the presence of septa, with higher prevalence in the middle region (16,18-20). On the other hand, some authors have demonstrated that the highest rate of prevalence of maxillary septa occurred in the posterior region (8,13,21). Rennie, Haffajee and Satyapal, in turn, noted that the majority of septa were localized in the anterior region of the maxilla (22). Khalighi Sigaroudi *et al*. observed a higher prevalence of maxillary sinus septa in the region of the molars and a higher association with perforation of the sinus membrane in this region (8).

Results with reference to the presence and absence of septa in the maxillary sinuses vary in different studies. Pommer *et al*. in a review study observed that the prevalence of septa was significantly lower in the Asiatic population (28,4%) (12), corroborating the findings of the present study, in which the majority of the Brazilian population (73,4%) revealed absence of septa. According to Talo Yildirim *et al*., the majority of patients examined presented no septa in the maxillary sinuses. However, in those in whom septa were present, the presence of only one of these structures was commonly noted, while no individual presented 3 septa (23). The result of the present study agrees that a large portion of the population has no presence of septa, thus also corroborating the studies of Hungerbühler *et al*., Talo Yildirim *et al*., Sakhdari *et al*. (9,15,23).

The present study demonstrated no significant difference in the presence of maxillary septa in relation to gender, agreeing with the results of Talo Yildirim *et al*. (23), Khalighi Sigaroudi *et al*. (8) and Marin *et al*. (7). Contrary to the studies of Orhan *et al*. (19), they noted a higher prevalence of septa in the male sex. Relative to side, no significance was also found, as reported by other authors (7,8,23).

As regards the fact of septa being complete or incomplete, Dragan *et al*. observed that 98% of dentate patients and 96% of edentulous patients presented complete septa, while 2% of dentate patients and 4% of edentulous patients presented incomplete septa (20). The data of the present research demonstrated that the majority of individuals with the presence of septa in the maxillary sinus presented the incomplete type, corroborating the studies of Dragan *et al*. and Pommer *et al*. in spite of not being a datum with statistical significance (12,20).

Regarding to the measurement of the sagittal height of the septa, this study presents higher mean values (7.9 +/- 3.0 mm) than showed by Talo Yildirim *et al*. (4.6 +/- 2.5 mm) (23). Sakhdari *et al*. and Kocak *et al*. (2019) demonstrated a mean height measure similar to the one shown in this research, corroborating with the results of the present study (9,24). Furthermore, it was also evaluated in this work the frontal and axial section height, which was not assessed in the similar researches.

In view of innumerable results of prevalence of maxillary septa in different populations, it became relevant to study these in a Brazilian population, seeing that the presence of maxillary septa may influence the success of a surgical procedure. We believe that the theme of this study will contribute to performing surgical planning, by avoiding intercurrences during the procedure. Therefore, conducting this study has collaborated by contributing fundamental information about septa in maxillary sinuses in a Brazilian population and making it available to Dental Professionals. Furthermore, we emphasize that this type of study may serve as a basis for further researches to be conducted in this area.
